# Supplementary material for: Cellular mechanotransduction of human osteoblasts in microgravity
Source: NPJ Microgravity. 2024 Mar 21;10:35. doi: 10.1038/s41526-024-00386-4 (PMC10957960; doi:10.1038/s41526-024-00386-4)
Supplement: Supplementary file 1 — Supplementary material [file 41526_2024_386_MOESM1_ESM.pdf]

Cellular mechanotransduction of human osteoblasts in microgravity

Nadab H. Wubshet[1,#], Grace Cai[2,#], Samuel J. Chen[1], Molly Sullivan[3], Mark Reeves[3], David Mays[3], Morgan Harrison[3], Paul Varnado[3], Benjamin Yang[4], Esmeralda Arreguin-Martinez[1], Yunjia Qu[4], Shan-Shan Lin[2], Pamela Duran[4], Carlos Aguilar[4], Shelby Giza[3], Twyman Clements[3,*], Allen P. Liu[1,2,4,5,6 *]

[1] Department of Mechanical Engineering, University of Michigan, Ann Arbor, MI, 48109, USA
[2] Applied Physics Program, University of Michigan, Ann Arbor, MI, 48109, USA
[3] Space Tango, Lexington, KY, 40505, USA
[4] Department of Biomedical Engineering, University of Michigan, Ann Arbor, MI, 48109, USA
[5] Department of Biophysics, University of Michigan, Ann Arbor, MI, 48109, USA
[6] Cellular and Molecular Biology Program, University of Michigan, Ann Arbor, MI, 48109, USA

[#] Equal contribution
* Corresponding authors: Twyman Clements, tclements@spacetango.com, Allen P. Liu, allenliu@umich.edu

Supplemental Figures

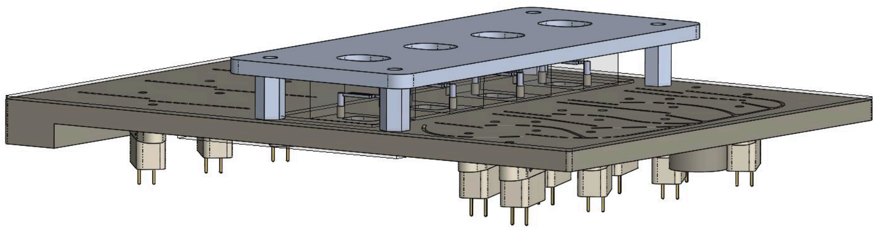

Supplemental Figure 1. CAD drawing of the fluidic manifold with a compression plate for holding down the microfluidic chips.

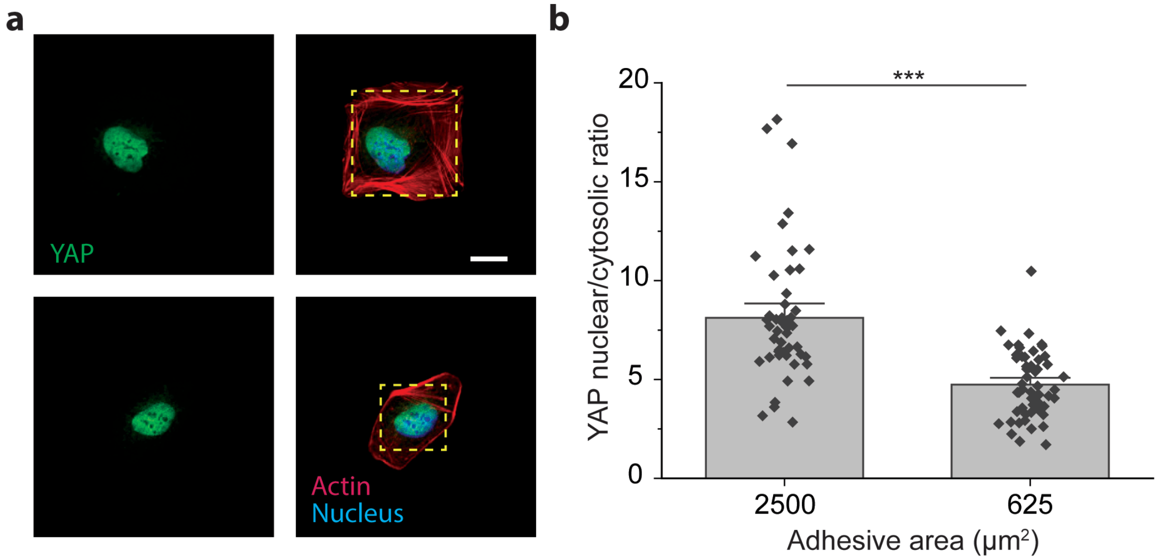

Supplemental Figure 2. YAP translocates to cytosol at low cell tension. A) Representative immunofluorescence images of human osteoblasts (hFOBs), on large (2500 μm^2) and small (625 μm^2) micropatterns, stained for F-actin, DNA, and YAP. Scale bar is 20 μm. **B)** Quantification of YAP nuclear/cytoplasmic ratio of individual hFOBs from three independent experiments. Error bars denote standard error of the means. *** $p < 0.001$.

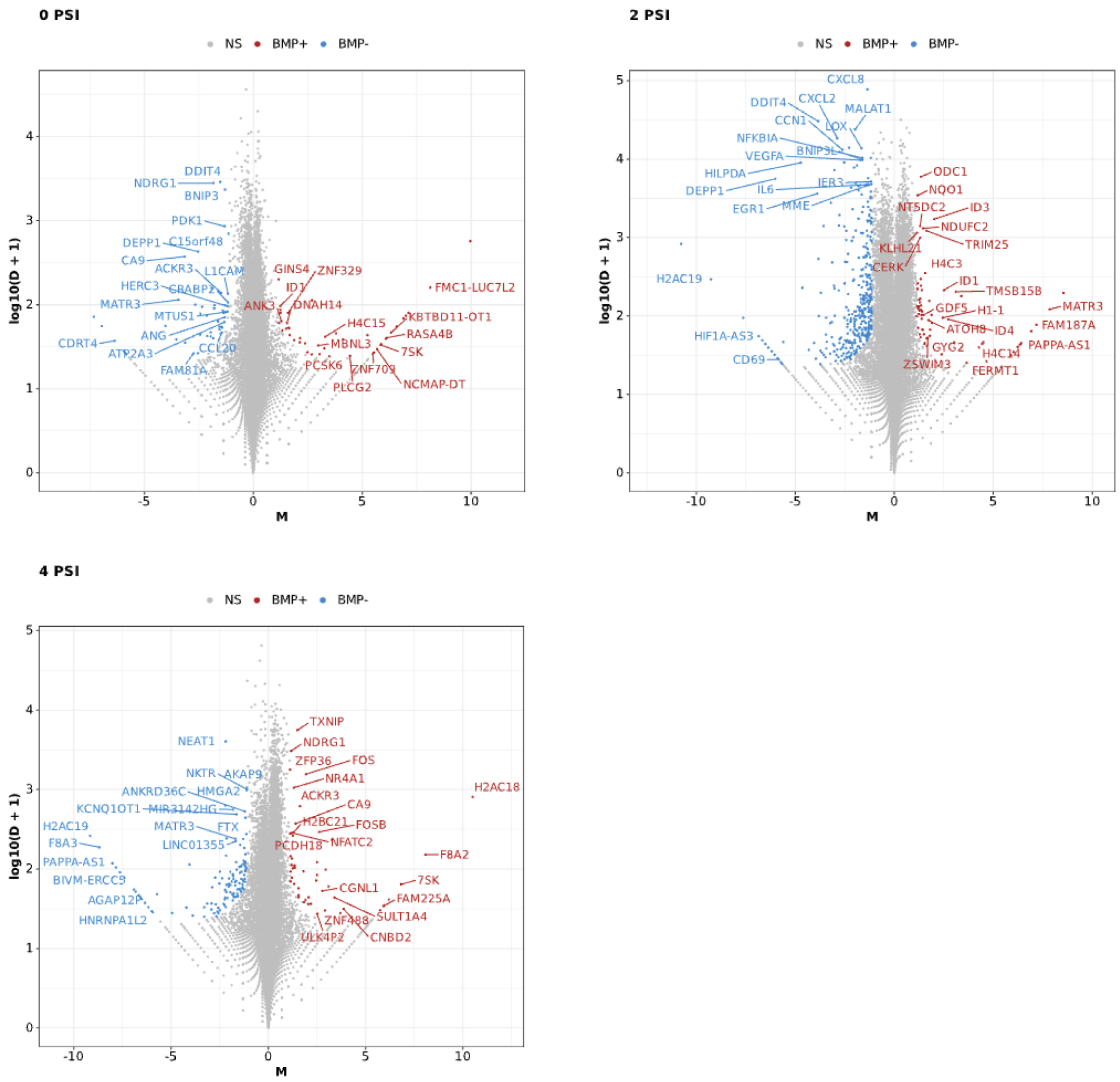

Supplemental Figure 3. Summary M-D plots showing differences in expression between BMP2 treated and untreated samples within each psi group. M values indicate log2 ratios between conditions. D values indicate the difference in counts between conditions. Significant genes are identified as those that are >90% likely to be differentially expressed compared to a null distribution.

Supplemental Tables

Supplemental Table 1. Timeline of the ISS experiment on microfluidic aspiration of human osteoblast (flown on SpX-27)

Date (2023)	Mission time days	Time from loading (hours)	Event (EST)
March 13	0		1:00 pm: final loading into CubeLab
March 14	1	28.5	5:30 pm: Handover to NASA
March 15	2	55.5	8:30 pm: Launch
March 16	3	66.5	7:30 am: Dock with ISS
March 17	4	90.5	7:50 am: Installed on ISS
March 17	4	95	10:30 am: Chip 2 seeding, pressurization, imaging/video
March 17	4	97	12:50 pm: Chip 3 seeding, pressurization, imaging/video
March 18	5	124.5	4:25 pm: Chip 4 seeding, pressurization, imaging/video
Payload remained powered to perform potential additional activities			
March 24	11	149	5:30 pm: Payload turned off

Supplemental Table 2. Well plate layout for the osteoblast spheroids pressurization experiment.

Well	1	2	3	4	5	6	7	8	9	10	11	12
Pressure (psi)	0	0	2	2	4	4	0	0	2	2	4	4
Ligand condition	BMP	None	BMP	None	BMP	None	BMP	None	BMP	None	BMP	None
Fixative	PFA	PFA	PFA	PFA	PFA	PFA	RNA Later	RNA Later	RNA Later	RNA Later	RNA Later	RNA Later

Supplemental Table 3. Timeline of the ISS experiment on osteoblast spheroid pressurization (flown on NG-18)

Date (2022)	Mission time days	Time from loading (hours)	Event (EST)
November 4	0		12:00 pm: Final loading into CubeLab
November 5	1	17	5:00 am: Handover to NASA
November 6	2	41	5:05 am: Launch attempt 1
November 7	3	66	5:30 am: Launch
November 8	4	95	11:18 am: Power shut off due to Cygnus solar panel malfunction
November 9	5	117	9:00 am Docked with ISS
November 9	5	123	3:30 pm: Power turned back on
November 10	6	139<br>151	7:04 am: Installed on to ISS<br>7:00 pm: Start serum starvation
November 11	7	162<br>164<br>165<br>168	5:45 am: Start BMP treatment<br>7:36 am: Start pressurization<br>8:45 am: Fixation start<br>11:33 am: Moved to cold stowage
